# Supplementary material for: Detection of cyberhate speech towards female sport in the Arabic Xsphere
Source: PeerJ Comput Sci. 2024 Jun 27;10:e2138. doi: 10.7717/peerj-cs.2138 (PMC11232602; doi:10.7717/peerj-cs.2138)
Supplement: Supplemental Information 1 [file peerj-cs-10-2138-s001.pdf]

Appendix A: Participants Demographics

Participants ID	Group	Participants Nationality	Participants Age
1	Sport Player	Saudi	18-25
2	Sport fan	Saudi	18-25
3	Sport Player	Saudi	18-25
4	Sport Player	Saudi	18-25
5	Sport Player	Saudi	26-30
6	Sport Player	Saudi	18-25
7	Sport Player	Saudi	31-36
8	Sport fan	Saudi	18-25
9	Sport Player	Saudi	18-25
10	Sport Player	Saudi	18-25
11	Sport Player	Saudi	26-30
12	Sport Player	Saudi	18-25
13	Sport Player	Saudi	18-25
14	Sport Player	Saudi	18-25
15	Sport Player	Saudi	18-25
16	Sport Player	Saudi	18-25
17	Sport fan	Saudi	37+
18	Sport Player	Saudi	18-25
19	Sport Player	Saudi	18-25
20	Sport Player	Saudi	18-25
21	Sport Player	Saudi	18-25
22	Sport Player	Saudi	26-30
23	Sport Player	Saudi	18-25
24	Sport Player	Saudi	18-25
25	Sport Player	Saudi	26-30
26	Sport fan	Saudi	18-25
27	Sport fan	Saudi	37+
28	Sport Player	Saudi	18-25
29	Sport Player	Saudi	18-25
30	Sport Player	Saudi	18-25
31	Sport Player	Saudi	26-30
32	Sport fan	Saudi	18-25
