# Supplementary material for: Detection of cyberhate speech towards female sport in the Arabic Xsphere
Source: PeerJ Comput Sci. 2024 Jun 27;10:e2138. doi: 10.7717/peerj-cs.2138 (PMC11232602; doi:10.7717/peerj-cs.2138)
Supplement: Supplemental Information 2 [file peerj-cs-10-2138-s002.pdf]

Appendix A: Interview Questions Study

Participant Information

1.What is your role in sports? Are you a player, a sports practitioner, a fan, or a follower of women's sports?

2.Select your age from the following options:
- 18-25
- 26-30
- 31-36
- 37 and above

Interview Questions

1.Do you use social media to follow women's sports or participate in discussions about women’s sports? If yes, please specify which platforms.

2.What kind of activities do you engage in on social media related to women's sports?

3.Have you experienced specific types of hate speech or online cyberbullying that you believe are directed at you as a female athlete or female supporter/fan? If yes, please provide examples?

4.Have you noticed any differences in how male athletes are treated on social media, specifically on Twitter, compared to female athletes? If yes, please provide some examples.

5.Is there a specific online group that promotes hatred towards women's sports or their followers?

6.Has dealing with hatred on social media, particularly on Twitter, affected your mental health, overall well-being, or your love for sports? If yes, how has it affected you? 7.Would you like to share any information that could help us with our research?
