# Supplementary material for: Detection of cyberhate speech towards female sport in the Arabic Xsphere
Source: PeerJ Comput Sci. 2024 Jun 27;10:e2138. doi: 10.7717/peerj-cs.2138 (PMC11232602; doi:10.7717/peerj-cs.2138)
Supplement: Supplemental Information 6 [file peerj-cs-10-2138-s006.pdf]

<u>بيانات المشاركة في الدراسه:</u>

1. ما هو دورك الرياضي هل انتي لاعبه أو ممارسه للرياضة أو مشجعه ومتابعه للرياضه النسائيه ؟
2. اختيار عمرك من الخيارات التاليه :
   - 18-25
   - 26-30
   - 31-36
   - 37-وأكثر

<u>أسئلة المقابلة:</u>

1. هل تستخدمين مواقع التواصل الأجتماعي للمشاركه أو متابعه مايخص الرياضة النسائيه؟ إذا انعم ارجو ذكرها
2. ما هو نوع النشاط التي تمارسه عبر مواقع التواصل الاجتماعي؟
3. هل لاحظت أي اختلافات في طريقة معاملة الرياضيين الذكور عبر مواقع التواصل الاجتماعي تحديدا (تويتر) مقارنة بالرياضيين الإناث؟ إذا نعم اذكر بعض الأمثله؟
4. هل واجهت أي أنواع محددة من الكراهية أو المضايقات الإلكترونية التي تعتقد أنها تستهدفك كرياضية؟
5. إذا اجابتك بنعم اذكر مثال؟ وماهي أكثر الوسائل التي تتلقون فيها مضايقات بكثرة؟ وهل يوجد تفاعل مع أو ضد هذه المضايقات؟
6. هل يوجد فئة معينة تبث أي نوع كراهية لممارسة المراءة الرياضة او متابعتها؟
7. هل أثر التعامل مع الكراهية عبر مواقع التواصل الاجتماعي تحديدا (تويتر) على صحتك العقلية أو رفاهيتك العامة أو حبك للرياضة؟ إذا نعم كيف كان التاثير؟
8. هل واجهت مشكلة مع والديك أو الأشخاص المقربين منك في تقبل أنك رياضية أو لديك اهتمامات رياضية؟ إذا إجابتك بنعم ما هو السبب؟
9. هل تعتقد أن هناك وعيا كافيا حول مخاطر الكراهية السيبرانية تجاه الرياضيات من النساء أو مشجعات الرياضه؟
10. هل تود مشاركه اي معلومة قد تساعدنا في البحث ؟
